# Electroacupuncture Modulates Programmed Cell Death 1 Ligand 1 on Peripheral and Central Nervous Systems in a Mouse Fibromyalgia Pain Model

**DOI:** 10.3390/biomedicines13020396

**Published:** 2025-02-06

**Authors:** Huan-Chin Lin, Hsin-Cheng Hsu, Hsien-Yin Liao, Arbee L.P. Chen, Yi-Wen Lin

**Affiliations:** 1 College of Chinese Medicine, Graduate Institute of Acupuncture Science, China Medical University, Taichung 404328, Taiwan; peishiuan1010@gmail.com; 2 Department of Traditional Chinese Medicine, Feng Yuan Hospital, Ministry of Health and Welfare, Taichung 420255, Taiwan; 3 Department of Traditional Chinese Medicine, China Medical University Hsinchu Hospital, China Medical University, Hsinchu 302056, Taiwan; 002762@tool.caaumed.org.tw; 4 College of Chinese Medicine, School of Post-Baccalaureate Chinese Medicine, China Medical University, Taichung 404328, Taiwan; 017215@tool.caaumed.org.tw; 5 Department of Computer Science and Information Engineering, Asia University, Taichung 413305, Taiwan; 6 Chinese Medicine Research Center, China Medical University, Taichung 404328, Taiwan

**Keywords:** electroacupuncture, fibromyalgia, PD-L1, TRPV1, spinal cord, somatosensory cortex

## Abstract

**Background:** Fibromyalgia, a chronic condition that causes long-lasting pain over several months, is a global medical issue with both personal and societal implications. It is one of the hardest types of pain to heal, given the lack of objective parameters for diagnosis and progression evaluation. The main symptoms of fibromyalgia are long-lasting widespread pain alongside with anxiety, fatigue, sleep disorders, cognitive dysfunction, and obesity. Programmed cell death 1 ligand 1 (PD-L1) has been used as a target in cancer immunotherapy. It can inhibit acute and chronic pain by suppressing nociceptive neuron activity via PD-1 receptors. **Methods**: The current study aimed to investigate the role of PD-L1/PD1 in a mouse fibromyalgia pain model. Mice were exposed to intermittent cold stress (ICS) to produce a murine fibromyalgia model characterized using von Frey and Hargreaves tests. **Results**: The ICS-induced mice fibromyalgia pain model showed mechanical (2.26 ± 0.18 g) and thermal (4.36 ± 0.31 s) hyperalgesia. Nociceptive responses could be relieved with electroacupuncture, intracerebral PD-L1 injection, or *Trpv1* deletion. We also identified a lower PD-1 level in the dorsal root ganglion, spinal cord, thalamus, and somatosensory cortex. In contrast, levels of pain-related kinases increased after fibromyalgia induction, an effect which could be reversed by EA, PD-L1, or *Trpv1* deletion. **Conclusions**: Our findings shed light on the contribution of PD-L1/PD1 to EA and fibromyalgia pain, indicating its potential as a treatment target for fibromyalgia.

## 1. Introduction

Programmed cell death 1 ligand 1 (PD-L1) is reported to suppress T cell function and induce immune tolerance via its receptor, PD-1 (1). Anti-PD1 and -PD-L1 treatments have been used for treating neuropathic or cancer pain [1,2]. However, it is unclear whether PD-L1/PD-1 can regulate chronic pain through immune neuronal modulation. Nociceptors appear to share characteristics with immune cells, as the brain not only responds to cytokines but can also regulate neural function through cell receptors. PD-L1/PD-1 is crucial as an immune mediator in the brain but also in chronic pain [1,2]. Current treatment approaches for fibromyalgia face multiple problems, ranging from cost-effectiveness to sustainability and target specificity. Furthermore, current clinical applications for general pain management include opioid, non-steroidal anti-inflammatory drugs (NSAIDS), COX-1 and COX-2 inhibitors, and sodium channel blockers, although the financial limitations, availability, and accessibility are all valid concerns alongside the plethora of side effects resulting from these treatments [3]. Fibromyalgia is a clinical challenge for many doctors, and its treatment is characterized by a supportive effort incorporating pharmaceutical and non-pharmaceutical medicines due to its unclear pathophysiology. Acupuncture was able to relieve fibromyalgia pain, stiffness, anxiety, and insomnia. However, it remains unclear whether neurons express functional PD-1 receptors in chronic pain, especially in relation to electroacupuncture (EA).

Transient receptor potential V1 (TRPV1) has been implicated in chronic pain, inflammation, cancer, and immunity [4,5]. It has been used as a drug target for various pain conditions, whereas a target for TRPV1 structure, agonists, and mechanisms is urgently needed [5,6]. TRPV1 activation mediates the contribution of phosphorylated phosphoinositide 3-kinase (pPI3K), phosphorylated Akt (pAkt), and phosphorylated mammalian target of rapamycin (pmTOR) in pain modulation, indicating a crucial role in central sensitization associated with fibromyalgia pain in both the peripheral and central nervous systems [7,8,9,10]. Furthermore, the epsilon isoform of protein kinase C (PKCɛ) has been considered as a transient insult that reliably delivers hyperalgesia to the nociceptors, resulting in long-lasting central sensitization, an essential factor in fibromyalgia pain. Mitogen-activated protein kinase (MAPK) is reportedly involved in inflammation and pain signaling in a pathway involving extracellular signal-regulated protein kinase, protein kinase p38 (p38), and c-Jun N-terminal kinase/stress-activated protein kinase (JNK) [11,12]. All of these elements increase their levels during nociception, a process associated with painful sensations, neuronal plasticity, central sensitization, and function ability of certain cognitive aptitudes [13,14]. PI3K-Akt-mTOR signaling appears to be associated with changes in the nociceptive response at both peripheral and central levels for its role in central sensitization. Moreover, nuclear factor kappa-light-chain-enhancer of activated B cells (NF-kB) has been found to be involved in fibromyalgia pain development; suppressing its expression has been associated with therapeutic benefits for pain [15,16].

Fibromyalgia pain is highly related to physiological and psychological issues. Characterized by a chronic widespread pain in the body, it is a disease representative of a serious growing issue including subjective, healthcare, economic, and societal problems. The incidence of fibromyalgia affects about 2–8% of the population, occurring often in women who suffer from tension headaches, irritable bowel syndrome, anxiety, and/or depression. Furthermore, women show lower pain thresholds and more severe symptoms than men [17,18]. Presently, there are few medicinal treatments for fibromyalgia, and those that exist can only relieve symptoms. Nutritional support, psychological treatment, exercise, and acupuncture are recommended. The pathogenesis of the disease is unknown. Peripheral and central sensitization may result in abnormal pain perception [19,20]. Inflammatory cytokines can influence neural networks during the interaction of the nervous system with immune cells, causing increased neuronal sensitization as well as neuroinflammation [21,22,23].

Acupuncture, a main component of traditional Chinese medicine, consists of inserting a steel needle into specific local acupoints and is reported to relieve many diseases and disorders, being especially useful in pain management. Traditional Chinese medicine describes acupuncture as a practice for balancing the chi to flow through the body meridians to improve health. In contrast, in Western medicine, acupuncture is supposed to stimulate peripheral nerves via connective tissue and muscle to manage symptoms. One study found that EA on the ST36 acupoint could activate the vagal–adrenal axis in mice via optogenetic stimulation of nerve terminals [24], while another report described a novel anti-inflammatory effect of EA in a mouse sepsis model [25]. We previously demonstrated that EA can trigger the release of adenosine triphosphate, interleukin-1β, interleukin-6, glutamate, substance P, and histamine at local acupoints [26]. Furthermore, EA has been reported to relieve Parkinson’s disease [27,28], manage body weight [29,30], decrease several pain syndromes in mice models, possibly by decreasing inflammatory cytokines such as interleukins, TNF-α, and IFN-γ in mouse plasma [16,31,32].

Based on the above, in the current study, we aimed to verify the hypothesis that fibromyalgia pain is associated with altered PD-L1/PD1 and related molecules in a mouse fibromyalgia model. In addition, we wanted to evaluate whether EA’s analgesic effect in both mechanical and thermal hyperalgesia is mediated by PD-L1 signaling. To address this issue, we developed a mouse model of fibromyalgia pain using intermittent cold stress (ICS). Consistent with our hypothesis, ICS caused significant mechanical and thermal hyperalgesia, which was attenuated by EA. PD-L1/PD1 and associated molecules reliably changed in ICS mice, being reversed by EA. Our data suggest that the analgesic effect of EA is related to PD-L1/PD1 in the dorsal root ganglion, spinal cord, thalamus, and somatosensory cortex. We found that EA modulates the PD-L1/PD1 signaling pathway, thus signifying new probable beneficial goals for treating fibromyalgia pain.

## 2. Materials and Methods

### 2.1. Mice and FM Pain Initiation

For the experiments, we used 8–12-week-old female C57B/L6 wild-type mice (18–20 g) sourced from BioLasc Taiwan Ltd. (Yilan, Taiwan) and housed in a specific pathogen-free environment. All mice were included for experiments without exclusion. After their arrival, mice were housed in cages under a 12 h light/dark cycle (light from 6 a.m. to 6 p.m.) at room temperature (25 °C) with 60% humidity. We performed statistical analysis to estimate the sample size; nine mice per group was considered the minimum number required for a significance α level of 0.05 and a power of 80%. Animal experiments were approved by the Institute of Animal Care and Use Committee of China Medical University (Permit no. CMUIACUC-2023-071), Taiwan, following the *Guide for the Care and Use of Laboratory Animals* (National Academy Press, Washington, DC, USA). The experimental researchers were blinded in group allocation and data analysis. Mice were randomly divided into five groups: normal (normal), cold stress-induced fibromyalgia, cold stress-induced fibromyalgia with EA (fibromyalgia + EA), cold stress-induced fibromyalgia with PD-L1 intracerebral injection (fibromyalgia + PD-L1), and cold stress-induced fibromyalgia in *Trpv1^−/−^* mice (fibromyalgia + *Trpv1^−/−^*). To create the fibromyalgia model, mice were placed at 4 °C, while the normal group was consistently maintained at 25 °C. At 10 a.m. the following day, fibromyalgia mice were moved to 25 °C for 30 min before being returned to 4 °C for another 30 min. This process was performed for 6 h until 4 p.m. before they were replaced again overnight, beginning at 4 p.m., during the first 3 days.

### 2.2. Electroacupuncture

Mice were anesthetized with 5% isoflurane for induction and maintained at 1% isoflurane for inhalation. Dual 1” steel acupuncture needles (32G, Yu Kuang Chem. Ind. Corp., Taipei, Taiwan) were implanted bilaterally into the mouse ST36 acupoint, located 3–4 mm under the patella, between the fibula and tibia, on the anterior side of the anterior tibial muscle. We delivered 1 mA strength, 2 Hz frequency, and 150 μs continuous square pulses for 20 min using an electronic Trio 300 stimulator (Ito, Japan). EA produced minor observable muscle twitching around the acupoint. EA was performed thrice from day 3 to day 4 after ICS.

### 2.3. Nociceptive Behavior Tests

Mechanical pain and thermal pain were measured three times on day 0, day 3, and day 4 before and after ICS. The pain threshold on the day before ICS induction was considered as the baseline. Mice were first placed in plexiglass boxes directly above a steel mesh in a dark, noiseless, room temperature location to keep them calm and allow them to adjust to the new environment. When mice were not moving, standing, sleeping, scratching, or grooming, the von Frey filament test was performed three times per session at 10 min intervals (IITC Life Science Inc., Burbank, Victory Blvd Woodland Hills, CA, USA). Next, the Hargreaves test was performed to measure thermal latency. The test started after 30 min of acclimation. An IITC Plantar Analgesia Meter (IITC Life, Sciences, SERIES8, Model 390G, Burbank, Victory Blvd Woodland Hills, CA, USA) determined the withdrawal latency of the mouse foot over radiant thermal light. The device was set to self-cut at 20 s to avoid damaging the hindpaws.

### 2.4. Western Blot

The dorsal root ganglion, spinal cord, thalamus, and somatosensory cortex were removed for extracting proteins. Brain samples were put on ice and then moved to a − 80°C refrigerator. Proteins were further nurtured in a cold radioimmunoprecipitation buffer. The extracted proteins were subjected to 8% SDS-Tris glycine gel electrophoresis and transmitted to a polyvinylidene difluoride membrane. The polyvinylidene difluoride was nurtured in 5% fat-free milk in TBS-T buffer (100 mM NaCl, 10 mM Tris pH 7.5, 0.1% Tween 20) and a primary monoclonal antibody against PD-1 (1:1000, cat #: CD-279, Invitrogen, Waltham, MA, USA), TRPV1 (1:1000, cat #: ACC-030, Alomone, Jerusalem, Israel), Nav1.7 (1:1000, cat #: ASC-008, Alomone, Israel), Nav1.8 (1:1000, cat #: ASC-028, Alomone, Israel), pPI3K (1:1000, cat #: PA5-28070, Invitrogen, Waltham, MA, USA), pAkt (1:1000, cat #: 9271, Cell Signaling Technology, Danvers, MA, USA), pmTOR (1:500, cat #: 05-1592, Merck, Darmstadt, Germany), pERK1/2 (1:1000, cat #: 36-8800, Invitrogen, Waltham, MA, USA), pCREB (1:1000, cat #: 06-519, Merck, Darmstadt, Germany), pNFκB (1:1000, cat #: ab86299, Abcam, Cambridge, UK), pp38 (1:1000, cat #: 44-684G, Invitrogen, Waltham, MA, USA), and pJNK (1:1000, cat #: 44-682G, Invitrogen, Waltham, MA, USA), which has high specificity to a single epitope and low cross-reactivity, with 1% bovine serum albumin for at least 1 h at room temperature. A peroxidase-conjugated anti-rabbit antibody or anti-mouse antibody (1:5000, cat #: A0545, Merck, Darmstadt, Germany) was utilized as a secondary antibody. Blot bands were imaged using a chemiluminescent substrate kit (PIERCE, Thermo Scientific, Waltham, MA, USA) and LAS-3000 Fujifilm (Fuji Photo Film Co., Ltd., Tokyo, Japan). The protein concentration of the bands was measured using NIH Image J 1.54h software (Bethesda, MD, USA). β-actin and α-tubulin were used as internal controls.

### 2.5. Immunofluorescence

Mice were anesthetized with 5% isoflurane and intracardially injected with 0.9% normal saline followed by 4% paraformaldehyde. Tissues were instantly excised and fixed with 4% paraformaldehyde at 4 °C for 3 days. Then, samples were incubated in 30% sucrose for post cryoprotection overnight at 4 °C, before being fixed in an optimal cutting temperature complex and quickly frozen in liquid nitrogen before storage at −80 °C. Frozen tissues were then chipped into 20 μm slices and placed on glass slides. The slices were next fixed in 4% paraformaldehyde and then nurtured in blocking solution with 3% BSA, 0.1% Triton X-100, and 0.02% sodium azide for 1 h. The slices were nurtured with the primary antibodies against PD-1 (1:200, cat #: CD-279, Invitrogen, Waltham, MA, USA), TRPV1 (1:200, cat #: ACC-030, Alomone, Israel) and prepared in 1% BSA solution overnight. Then, samples were incubated with the secondary antibodies (1:500) 488-conjugated AffiniPure donkey anti-rabbit IgG (H + L) and 594-conjugated AffiniPure donkey anti-goat IgG (H + L) (cat #: A17041, Thermo Scientific, Waltham, MA, USA) for 2 h at room temperature before fixation with coverslips for immunofluorescence visualization.

### 2.6. Intracerebroventricular Injection

Mice were anesthetized with isoflurane with immobilized heads in a stereotaxic frame to allow a cannula to be implanted at the ventricle. The stereotaxic cannula (23 gauge 2 mm stainless steel, Doric, QC, Canada) was then placed at 0.5 mm in the anteroposterior axis, around 1 mm in the mediolateral axis, and about 2.5 mm in the dorsoventral axis under the cortical surface and fixed at the skull with dental cement. Afterwards, the cannula was inserted and connected to a Hamilton syringe through a PE tube (PE10, Portex, Kent, UK). A total of 5 μg of PD-L1 (5 μL/ventricle) was injected over a period of 5 min using a syringe pump (KD Scientific, Shanghai, China). After inoculation, the cannula was left at the ventricle for an extra 2 min to allow the PD-L1 to diffuse.

### 2.7. Statistical Analysis

Statistical analysis was performed using SPSS 21.0. All statistical data are presented as the mean ± standard error (SEM). A Shapiro–Wilk test was performed to examine the normality of the results. Statistical differences among groups were tested using an ANOVA test, followed by a post hoc Tukey’s test. *p* < 0.05 was considered to indicate statistical significance.

## 3. Results

### 3.1. EA, PD-L1 Injection, or Trpv1 Deletion Care for FM Pain in a Mouse Model

**Figure 1:**
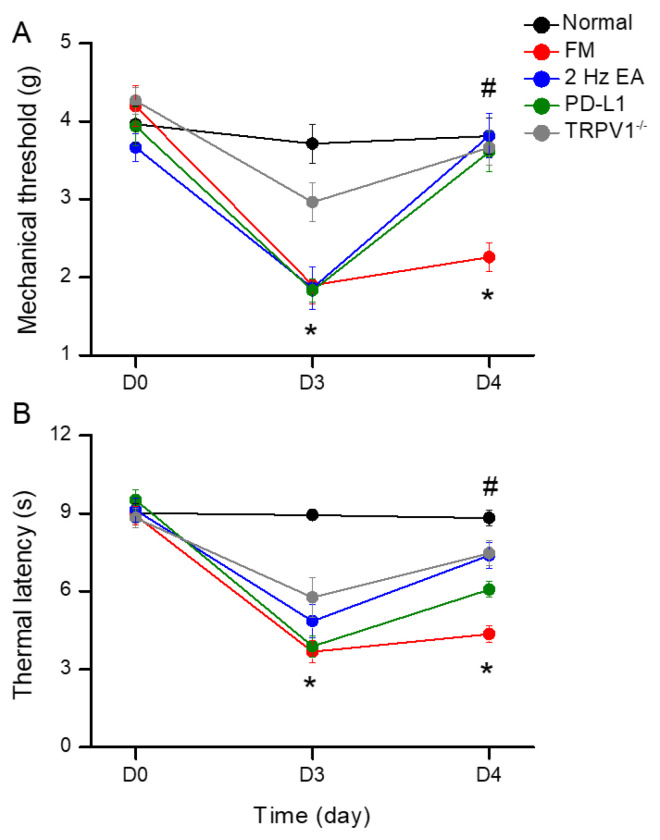
Mechanical and thermal hypersensitivity of mice in all groups: (**A**) von Frey filament test indicating the mechanical threshold; (**B**) Hargreaves test estimating thermal latency. The FM induction significantly augmented hyperalgesia in mechanical and thermal sensitivity. The noteworthy reducing threshold and latency were improved in 2 Hz electroacupuncture (EA), PD-L1, and *Trpv1^−/−^* mice. * significant difference to normal group. ^#^ significant difference to fibromyalgia (FM) group. *n* = 9 per group.

We induced fibromyalgia pain in normal and *Trpv1*^−/−^ mice through ICS induction. In normal mice, mechanical threshold and thermal latency were normal on day 3 to day 4 (Figure 1A). Due to ICS, mechanical and thermal hyperalgesia increased, and the levels were maintained for 2 days, as indicated by von Frey and Hargreaves tests (Figure 1A,B, red circles, 2.26 ± 0.18 g, and 4.36 ± 0.31 s, * *p* < 0.05, *n* = 9). Treatment with 2 Hz EA significantly attenuated both mechanical and thermal hyperalgesia (Figure 1A,B, blue circles, 3.81 ± 0.28 g and 7.38 ± 0.48 s, ^#^ *p* < 0.05, *n* = 9). Intracerebral PD-L1 injections relieved mechanical and thermal hyperalgesia (Figure 1A,B, green circles, 3.61 ± 0.26 g and 6.08 ± 0.31 s, ^#^ *p* < 0.05, *n* = 9). TRPV1 loss improved both mechanical and thermal hyperalgesia on day 4 after ICS induction (Figure 1A,B, gray circles, 3.66 ± 0.22 g and 7.48 ± 0.48 s, ^#^ *p* < 0.05, *n* = 9).

### 3.2. EA Controlled PD1-TRPV1 Pain Signaling in the Peripheral DRG of FM Mice

**Figure 2:**
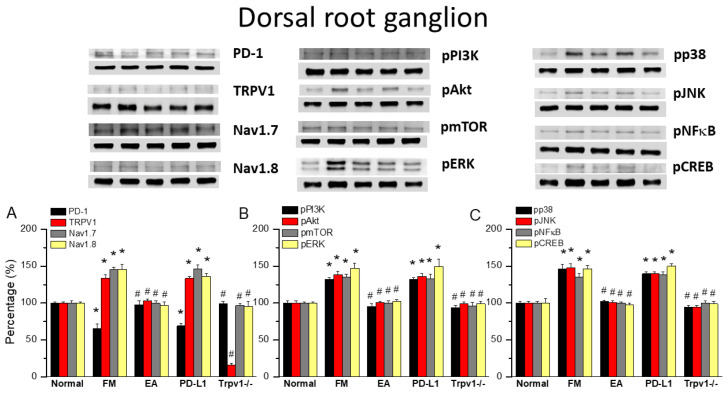
Changing levels of PD-1 and related molecules in the dorsal root ganglion. Western blot data with five lanes of proteins for normal, fibromyalgia (FM), FM + EA, PD-L1, and *Trpv1*^−/−^ groups: (**A**) PD-1, TRPV1, Na_v_1.7, and Na_v_1.8; (**B**) pPI3K, pAkt, pmTOR, and pERk; and (**C**) pp38, pJNK, pNF-κB, and pCREB levels that were increased in the FM group and were attenuated in 2 Hz electroacupuncture (EA) or *Trpv1*^−/−^ but not PD-L1 mice. * Significant difference to normal. ^#^ Significant difference to fibromyalgia (FM). *n* = 6.

Initially, we aimed to determine whether PD1 and TRPV1 affect fibromyalgia pain in peripheral dorsal root ganglion areas. We used Western blot to define the valence associated with PD1-TRPV1 modulation. PD1 was expressed in the dorsal root ganglion, but its level was reduced with fibromyalgia (Figure 2A, 65.91 ± 6.23%, black column, * *p* < 0.05, *n* = 6). EA significantly augmented the reduction in PD1 in the mouse dorsal root ganglion after ICS induction (Figure 2A, 97.99 ± 5.11%, black column, ^#^ *p* < 0.05, *n* = 6). Intracerebral PD-L1 administration did not change the expression of the PD1 receptor in the dorsal root ganglion (Figure 2A, 68.98 ± 3.46%, black column, * *p* < 0.05, *n* = 6). To establish a relationship between PD1 and TRPV1, we subjected *Trpv1*^−/−^ mice to ICS induction. Our results indicated that PD1 receptors return to normal levels in fibromyalgia model mice (Figure 2A, 98.82 ± 3.60%, black column, ^#^ *p* < 0.05, *n* = 6). TRPV1 was presented in the dorsal root ganglion, increasing with fibromyalgia pain (Figure 2A, 133.89 ± 4.63%, red column, * *p* < 0.05, *n* = 6), while 2 Hz EA inhibited this overexpression (Figure 2A, 102.93 ± 2.57%, red column, ^#^ *p* < 0.05, *n* = 6). Furthermore, TRPV1 expression in the dorsal root ganglion was not attenuated by PD-L1 injection (Figure 2A, 133.46 ± 2.36%, red column, * *p* < 0.05, *n* = 6), being almost lost in *Trpv1*^−/−^ mice (Figure 2A, 16.47 ± 2.06%, red column, ^#^ *p* < 0.05, *n* = 6). Next, we verified the expression of associated nociceptive ion channels such as Na_v_1.7 and Na_v_1.8, potential targets for drug development in pain. Their protein levels increased with ICS and could be alleviated by 2 Hz EA. PD-L1 administration similarly attenuated Na_v_1.7 and Na_v_1.8 overexpression. Similar results were observed in *Trpv1*^−/−^ mice under ICS (Figure 2A, gray and yellow columns, * *p* < 0.05, *n* = 6).

We next aimed to identify TRPV1-related kinases in this model, namely components of the pPI3K-pAkt-pmTOR axis. As shown in Figure 2B, the expression of pPI3K-pAkt-pmTOR increased in the dorsal root ganglion of fibromyalgia mice. The Western blot results indicated that these protein levels were downregulated after EA treatment. Similar results were observed with PD-L1 injection and in *Trpv1*^−/−^ mice (Figure 2B, individual column, *p < 0.05, *n* = 6). A noticeably higher expression of MAPK (pERK, pp38, and pJNK) kinases was observed in the dorsal root ganglion of fibromyalgia mice compared to the normal group, an effect inhibited by 2 Hz EA treatment. Similar results were obtained after PD-L1 injection and in *Trpv1*^−/−^ mice. Moreover, the expression of pNKκB and pCREB increased in the dorsal root ganglion of fibromyalgia mice compared to normal mice. Finally, 2 Hz EA, PD-L1 injection, and *Trpv1* deletion all reversed these changes (Figure 2B,C, individual column, * *p* < 0.05, *n* = 6).

### 3.3. EA at ST36 Diminished Cold Stress-Induced FM Pain Through TRPV1-CB1 in the SCDH

**Figure 3:**
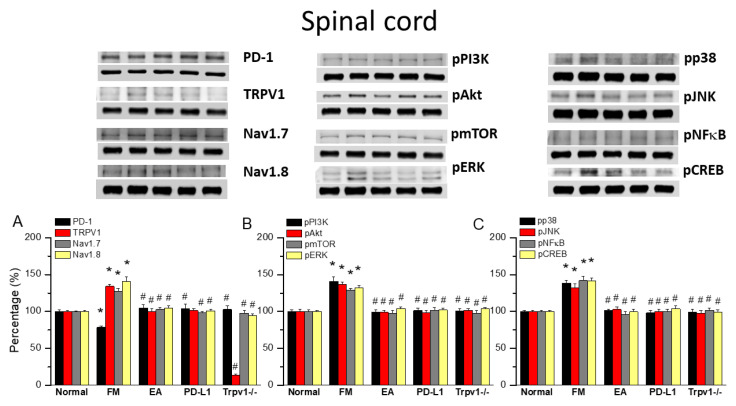
Changing levels of PD-1 and related molecules in the spinal cord. Western blot data with five lanes of proteins for normal, fibromyalgia (FM), FM + EA, PD-L1, and *Trpv1*^−/−^ groups: (**A**) PD-1, TRPV1, Na_v_1.7, and Na_v_1.8; (**B**) pPI3K, pAkt, pmTOR, and pERk; and **(C)** pp38, pJNK, pNF-κB, and pCREB levels that were increased in the FM group and were attenuated in 2 Hz electroacupuncture (EA), PD-L1, and *Trpv1*^−/−^ mice. * Significant difference to normal. ^#^ Significant difference to fibromyalgia (FM). *n* = 6.

To gain insights into the role of PD1/PD-L1 in the spinal cord of fibromyalgia mice, we applied Western blot to samples obtained at 2 days after ICS induction, the subacute stage of pain. PD-1 expression was normal in the normal group but significantly lower in the spinal cord of fibromyalgia mice (Figure 3A, 78.64 ± 2.11%, black column, * *p* < 0.05, *n* = 6), a decrease deepened by 2 Hz EA treatment (Figure 3A, 104.69 ± 4.80%, black column, ^#^ *p* < 0.05, *n* = 6). This pattern was also obtained with intracerebral PD-L1 injection (Figure 3A, 104.14 ± 5.67%, black column, ^#^ *p* < 0.05, *n* = 6) and in *Trpv1*^−/−^ mice (Figure 3A, 102.75 ± 5.43%, black column, ^#^ *p* < 0.05, *n* = 6). Interestingly, TRPV1 increased after fibromyalgia induction compared to controls (Figure 3A, 134.21 ± 2.45%, red column, * *p* < 0.05, *n* = 6). Moreover, 2 Hz EA not only relieved fibromyalgia pain but also decreased TRPV1 levels in the spinal cord (Figure 3A, 100.13 ± 3.32%, red column, ^#^ *p* < 0.05, *n* = 6), as did PD-L1 injection (Figure 3A, 101.16 ± 2.95%, red column, ^#^ *p* < 0.05, *n* = 6), and disappeared in the *Trpv1* knock out (Figure 3A, 13.66 ± 1.09%, red column, ^#^ *p* < 0.05, *n* = 6). The levels of nociceptive ion channels Na_v_1.7 and Na_v_1.8 increased after fibromyalgia induction, being decreased by 2 Hz EA, PD-L1 administration, and in *Trpv1*^−/−^ mice (Figure 3A, gray and yellow columns, * *p* < 0.05, *n* = 6).

With respect to pPI3K, pAkt, and pmTOR, their levels were increased in the spinal cord of FM mice but decreased by 2 Hz EA treatment, PD-L1 injection, and in *Trpv1*^−/−^ mice (Figure 3B, * *p* < 0.05, *n* = 6). Similar results were obtained for pERK, pp38, and pJNK. pNKκB and pCREB levels were increased in the spinal cord of fibromyalgia mice but decreased with 2 Hz EA, PD-L1 injection, and *Trpv1* knock out (Figure 3B,C, * *p* < 0.05, *n* = 6).

### 3.4. EA at ST36 Altered FM Pain and Regulated PD-1 Signaling Pathway in the Thalamus

**Figure 4:**
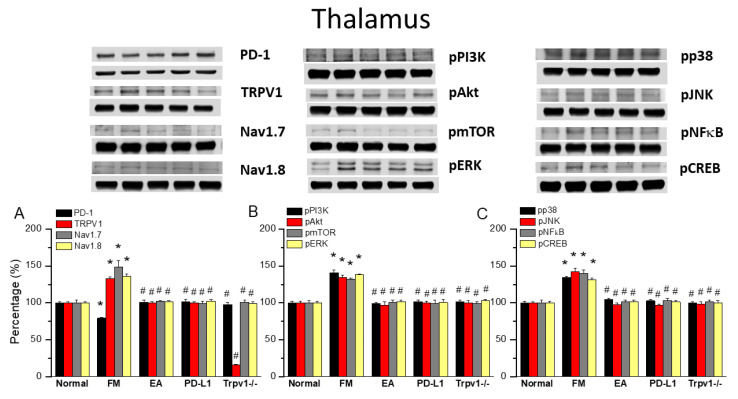
Changing levels of PD-1 and related molecules in the thalamus. Western blot data with five lanes of proteins for normal, FM, FM + EA, PD-L1, and *Trpv1*^−/−^ groups. (**A**) PD-1, TRPV1, Na_v_1.7, and Na_v_1.8; (**B**) pPI3K, pAkt, pmTOR, and pERk; and (**C**) pp38, pJNK, pNF-κB, and pCREB levels that were increased in the FM group and were attenuated in 2 Hz electroacupuncture (EA), PD-L1, and *Trpv1*^−/−^ mice. * Significant difference to normal. ^#^ Significant difference to fibromyalgia (FM). *n* = 6.

To observe the effects of PD-L1/PD1 on FM mice, we measured the expression levels of PD1 in the ascending pain pathway thalamus. After completing behavioral tests on day 4, we collected the thalamus and calculated the expression levels of previously mentioned proteins via Western blot. PD-1 levels were consistently abridged in thalamus of FM mice (Figure 4A, 79.07 ± 0.88%, black column, * *p* < 0.05, *n* = 6) and 2 Hz EA effectively increased them (Figure 4A, 100.83 ± 3.26%, black column, ^#^ *p* < 0.05, *n* = 6), as did intracerebral injection of PD-L1 (Figure 4A, 101.15 ± 3.39%, black column, ^#^ *p* < 0.05, *n* = 6). Conversely, the reduction in PD1 level was not observed in the thalamus of *Trpv1*^−/−^ mice (Figure 4A, 97.55 ± 3.04%, black column, ^#^ *p* < 0.05, *n* = 6). Consistent with earlier findings, TRPV1 levels increased on day 4 after ICS induction in the thalamus of FM mice compared with the normal group (Figure 4A, 132.78 ± 2.47%, black column, * *p* < 0.05, *n* = 6). Mice treated with 2 Hz EA showed decreased TRPV1 levels in the thalamus (Figure 4A, 100.09 ± 1.49%, red column, ^#^ *p* < 0.05, *n* = 6). Similar trends were also obtained with the intracerebral injection of PD-L1 (Figure 4A, 100.21 ± 1.72%, red column, ^#^ *p* < 0.05, *n* = 6) and the disappearance of *Trpv1*^−/−^ (Figure 4A, 15.97 ± 1.06%, red column, ^#^ *p* < 0.05, *n* = 6) mice, as well as for Na_v_1.7 and Na_v_1.8 (Figure 4A, gray and yellow columns, * *p* < 0.05, *n* = 6).

Similarly, pPI3K, pAkt, and pmTOR levels increased after ICS but decreased with 2 Hz EA, PD-L1 injection, and *Trpv1* knock out (Figure 4B, * *p* < 0.05, *n* = 6). FM pain also increased the expression of pERK, pp38, and pJNK in the thalamus, suggesting a vital function in ascending pain signaling. Moreover, 2 Hz EA treatment demonstrated a key therapeutic effect on FM pain through regulating these kinases. The same trends were observed after intracerebral PD-L1 injection and *Trpv1* knock out. Likewise, ICS resulted in pNFκB and pCREB overexpression, an effect reversed by 2 Hz EA, PD-L1 injection and *Trpv1* knock out (Figure 4B,C, * *p* < 0.05, *n* = 6).

### 3.5. TRPV1 and Related Factors Were Inhibited in the Somatosensory Cortex After ICS and Recovered by 2 Hz EA and Trpv1 Deletion

**Figure 5:**
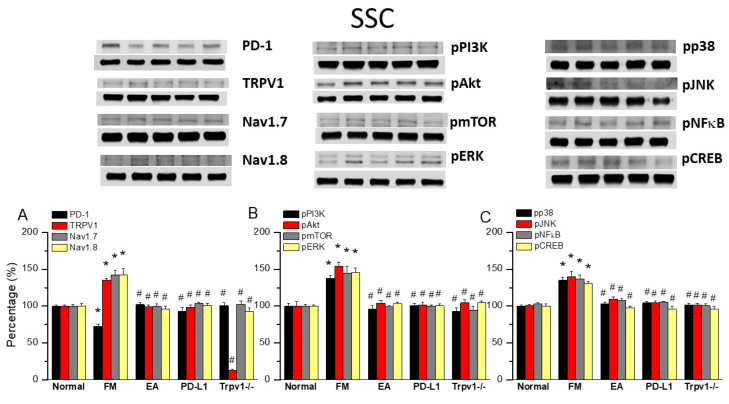
Changing levels of PD-1 and related molecules in the SSC. Western blot data with five lanes of proteins for normal, FM, FM + EA, PD-L1, and *Trpv1*^−/−^ groups: (**A**) PD-1, TRPV1, Na_v_1.7, and Na_v_1.8; (**B**) pPI3K, pAkt, pmTOR, and pERk; and (**C**) pp38, pJNK, pNF-κB, and pCREB levels that were increased in the FM group and were attenuated in 2 Hz electroacupuncture (EA), PD-L1, and *Trpv1^−/−^* mice. * Significant difference to normal. ^#^ Significant difference to fibromyalgia (FM). *n* = 6.

Finally, to see whether there is a difference in PD1 when determining the influence of ICS-induced fibromyalgia pain, we also aimed to identify whether EA could affect fibromyalgia pain signaling in the ascending pathway, so we dissevered the mice somatosensory cortex for protein analysis. ICS significantly abridged the expression of PD1 in the mice somatosensory cortex compared with normal mice (Figure 5A, 72.70 ± 3.25%, black column, * *p* < 0.05, *n* = 6). This diminution was reversed by 2 Hz EA (Figure 5A, 102.16 ± 2.42%, black column, ^#^ *p* < 0.05, *n* = 6), PD-L1 injection (Figure 5A, 92.94 ± 5.38%, black column, ^#^ *p* < 0.05, *n* = 6), and *Trpv1* deletion (Figure 5A, 100.87 ± 3.64%, black column, ^#^ *p* < 0.05, *n* = 6). Compared to controls, a significant increase in TRPV1, Na_v_1.7, and Na_v_1.8 levels was observed in fibromyalgia mice. This increase was reversed by 2 Hz EA, intracerebral PD-L1 injection, and *Trpv1* deletion (Figure 5A, * *p* < 0.05, *n* = 6). Similarly, pPI3K, pAkt, and pmTOR expression increased in fibromyalgia mice compared to normal mice, becoming significantly decreased by 2 Hz EA, intracerebral injection of PD-L1, and *Trpv1* deletion. Similar results were obtained for pERK, pp38, pJNK, pNFκB, and pCREB (Figure 5B,C, * *p* < 0.05, *n* = 6).

### 3.6. EA, Intracerebral PD-L1 Injection, or TRPV1 Loss Mitigated FM in the DRG or SSC

**Figure 6:**
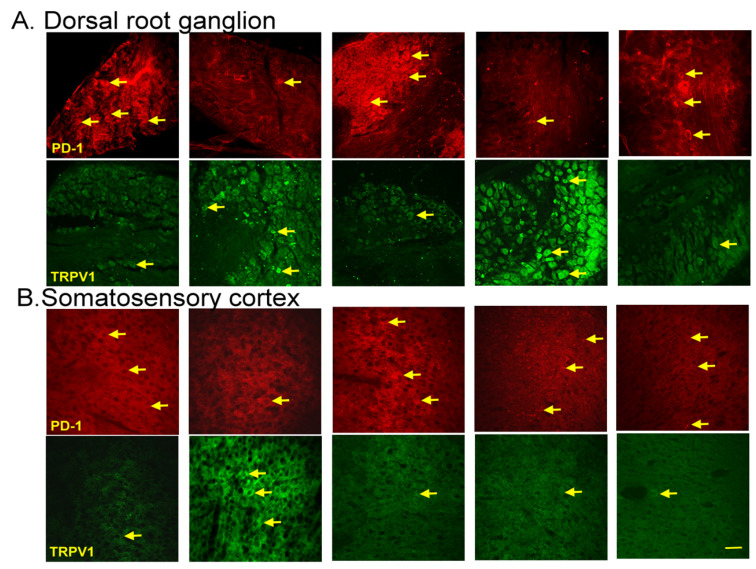
Immunofluorescence staining of PD-1 and TRPV1 to evaluate protein expression in the mouse (**A**) dorsal root ganglion and (**B**) somatosensory cortex. The yellow arrows indicate immune-positive signals. *n* = 3 for all groups. scale bars = 100 μm.

Immunofluorescence staining of the dorsal root ganglion for PD-1 showed lower PD-1 expression in the dorsal root ganglion of fibromyalgia mice than in normal mice, an effect reversed by 2 Hz EA, intracerebral injection of PD-L1, or *Trpv1* knock out. In contrast, TRPV1 protein levels were augmented in fibromyalgia mice, being attenuated by 2 Hz EA, PD-L1 injection, or TRPV1 loss (Figure 6A, yellow arrows, *n* = 3). Finally, we confirmed that PD-1 levels were decreased after ICS in the somatosensory cortex but increased after EA treatment, PD-L1 injection, or *Trpv1* deletion. Conversely, TRPV1 levels increased after fibromyalgia induction and were decreased by EA, PD-L1 injection, or *Trpv1* deletion (Figure 6B, yellow arrows, *n* = 3).

## 4. Discussion

To our knowledge, this is the first comprehensive study on the relationship between EA, fibromyalgia pain, and PD-L1/PD-1 signaling. ICS-induced fibromyalgia resulted in mechanical and thermal pain, as indicated by von Frey and Hargreaves tests on mice, an effect that can be reversed with 2 Hz EA, PD-L1 injection, or *Trpv1* deletion. We found that PD-L1/PD-1 signaling was attenuated in the peripheral dorsal root ganglion as well as in the central spinal cord, thalamus, and somatosensory cortex. We also demonstrated that levels of nociceptive TRPV1 and related molecules were increased after fibromyalgia induction. These tendencies could be reversed by 2 Hz EA, PD-L1 injection, or *Trpv1* deletion. However, intracerebral ventricular injection of PD-L1 could not attenuate TRPV1 overexpression at peripheral dorsal root ganglion sites, suggesting its advanced and subsequent levels. The turnover of these protein alterations in signaling protein kinases through the use of EA suggests that this management, especially its relationship with PD-L1/PD-1, can be used as an efficient beneficial intervention.

Chen et al. demonstrated that, in the dorsal root ganglion, PD-L1 can effectively prevent acute and chronic pain. An intraplantar PD-L1 injection induced anti-nociception in normal mice via PD-1, as well as blockage of PD-L1 cross-talk into mechanical allodynia. Gene deletion of *Pd1* displayed mechanical and thermal hypersensitivity. Furthermore, PD-1 activation in dorsal root ganglion nociceptive neurons was reported to induce Src homology 2 domain-containing tyrosine phosphatase-1 (SHP-1) phosphorylation to activate TREK2 K^+^ channels for pain relief [32]. Liu et al. demonstrated that PD-L1 can stimulate SHP-1 to decrease TRPV1 expression in the dorsal root ganglion, relieving mouse bone cancer pain. In addition, TRPV1 blockade reliably attenuated cancer pain-related hyperalgesia and PD-L1 analgesia, suggesting a crucial role of TRPV1 in PD-L1 signaling. They also reported that a conditioned SHP-1 knock out increased bone cancer pain and reduced PD-L1 inhibition of TRPV1 in peripheral dorsal root ganglion neurons [33].

Kong et al. demonstrated that PD-L1 was increased after spinal cord injury on microglia or macrophages at the injury site. Loss of PD-L1 in mice caused poorer quality of motor function and nociception with increased polarization of M1-like macrophages. They next determined that PD-L1′s effects after spinal cord injury occur by attenuating phosphorylation of p38 and pERK2 [34]. Shi et al. reported a crucial role of PD-L1/PD-1 in the trigeminal ganglia in a mouse migraine model after showing PD-L1/PD-1 immunoreactivity in normal trigeminal ganglia neurons. The levels of PD-L1/PD-1 were increased after migraine initiation. Inhibition of PD-1 amplified migraine-induced hyperalgesia was accompanied by increased calcitonin gene-related peptide, interleukin-1β, tumor necrosis factor α, interleukin-6, and interleukin-18 in the trigeminal ganglia of mice [35].

Tan et al. found that mechanical and thermal onsets were increased, as was the level of PD-L1, in late pregnancy. In addition, soluble PD-1 can significantly decrease the pain thresholds of late pregnancy mice. Moreover, interleukin-10 was visibly increased, and tumor necrosis factor α and interleukin-6 were attenuated in late pregnancy mice, suggesting the crucial role of PD-L1/PD-1 pathway in pregnancy-induced analgesia [36]. Recently, PD-L1/PD-1 activation has been found to attenuate nociceptor excitability and induce important pain-relieving effects, suggesting that it is a beneficial target for pain management. Zhao et al. revealed that H-20 can bind to PD-1 with micromolar affinity and later initiate SHP-1 phosphorylation in dorsal root ganglion neurons. Spinal H-20 administration showed an actual and longer analgesic effect in preclinical models without side effects [37]. A recent article indicated that EA can restore immune function in malignant tumor patients by increasing lymphocytes [38]. Wang et al. reported that EA can enhance the immunotherapy response in microsatellite-stable colorectal cancer [39]. We suggest that EA can increase lymphocytes and further increase the release of PD-L1 to treat fibromyalgia.

## 5. Conclusions

**Figure 7:**
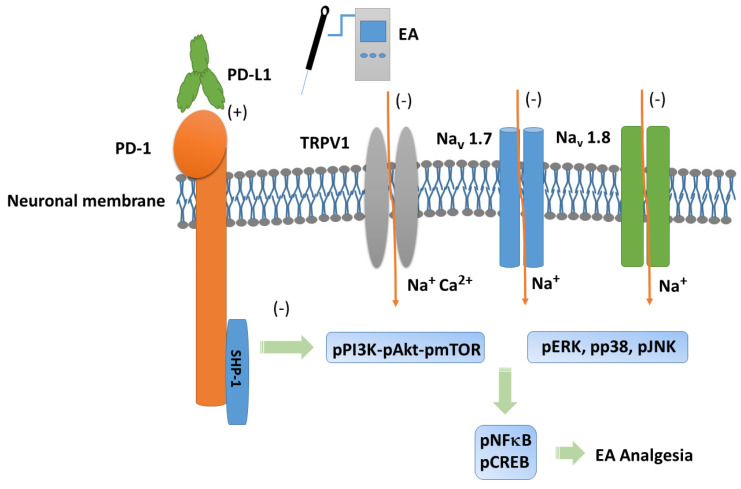
Programmed cell death 1 ligand 1 (PD-L1) signaling pathway with specific effect on PD-1. Abbreviations: EA = electroacupuncture; pERK = phosphorylated extracellular signal-regulated kinase; pp38 = phosphorylated protein kinase p38; pJNK = phosphorylated c-Jun N-terminal kinase; PD-L1 = programmed cell death 1 ligand 1; pPI3K = phosphorylated phosphoinositide 3-kinase; pAkt = phosphorylated Akt; pmTOR = phosphorylated mammalian target of rapamycin; TRPV1 = transient receptor potential V1; pNF-kB = phosphorylated nuclear factor kappa-light-chain-enhancer of activated B cells; pCREB = phosphorylated cAMP response element-binding protein. Orange arrows mean ion influx. Green arrows mean progress.

Our experiments provide evidence of necessary possessions of 2 Hz EA in mice fibromyalgia pain, especially via PD-L1/PD-1 and TRPV1 signaling pathways. We demonstrated the analgesic effect of EA on mouse fibromyalgia pain and its underlying mechanisms. We found that TRPV1 and related kinases were overexpressed in a mouse model of fibromyalgia pain in the dorsal root ganglion, spinal cord, thalamus, and somatosensory cortex. These effects were abolished by 2 Hz EA, PD-L1 injection, or *Trpv1* deletion. However, intracerebroventricular PD-L1 injection did not reduce TRPV1 overexpression at peripheral dorsal root ganglion sites, suggesting an ineffective long-term effect. Our results indicated the clinical relevance and application of EA in treating fibromyalgia pain. Future work should involve translating EA-based therapy to human FM patients. A schematic drawing of our findings is provided in Figure 7.

The limitation of this research was that we only examined the PD1 signaling pathway in female mice. We were not able to judge whether this conclusion can be obtained in male mice. We also cannot be certain about potential changes in EA effects depending on electrode placement or duration. Male mice or different parameters including EA duration or frequencies should be utilized in future work.

## Data Availability

The original contributions presented in this study are included in the article. Further inquiries can be directed to the corresponding authors.

## References

[1] Wang G., Kang X., Chen K.S., Jehng T., Jones L., Chen J., Huang X.F., Chen S.Y. (2020). An engineered oncolytic virus expressing PD-L1 inhibitors activates tumor neoantigen-specific T cell responses. Nat. Commun..

[2] Chen G., Kim Y.H., Li H., Luo H., Liu D.L., Zhang Z.J., Lay M., Chang W., Zhang Y.Q., Ji R.R. (2017). PD-L1 inhibits acute and chronic pain by suppressing nociceptive neuron activity via PD-1. Nat. Neurosci..

[3] Brown E.N., Pavone K.J., Naranjo M. (2018). Multimodal General Anesthesia: Theory and Practice. Anesth. Analg..

[4] Benitez-Angeles M., Morales-Lazaro S.L., Juarez-Gonzalez E., Rosenbaum T. (2020). TRPV1: Structure, Endogenous Agonists, and Mechanisms. Int. J. Mol. Sci..

[5] Koivisto A.P., Voets T., Iadarola M.J., Szallasi A. (2024). Targeting TRP channels for pain relief: A review of current evidence from bench to bedside. Curr. Opin. Pharmacol..

[6] Fernandez-Carvajal A., Fernandez-Ballester G., Ferrer-Montiel A. (2022). TRPV1 in chronic pruritus and pain: Soft modulation as a therapeutic strategy. Front. Mol. Neurosci..

[7] Fischer S.P.M., Brusco I., Brum E.S., Fialho M.F.P., Camponogara C., Scussel R., Machado-de-Avila R.A., Trevisan G., Oliveira S.M. (2020). Involvement of TRPV1 and the efficacy of α-spinasterol on experimental fibromyalgia symptoms in mice. Neurochem. Int..

[8] Yuksel E., Naziroglu M., Sahin M., Cig B. (2017). Involvement of TRPM2 and TRPV1 channels on hyperalgesia, apoptosis and oxidative stress in rat fibromyalgia model: Protective role of selenium. Sci. Rep..

[9] Hsiao I.H., Lin Y.W. (2022). Electroacupuncture Reduces Fibromyalgia Pain by Attenuating the HMGB1, S100B, and TRPV1 Signalling Pathways in the Mouse Brain. Evid.-Based Complement. Altern. Med..

[10] Liao H.Y., Lin Y.W. (2021). Electroacupuncture reduces cold stress-induced pain through microglial inactivation and transient receptor potential V1 in mice. Chin. Med..

[11] Zhou X., Zhang Y.C., Lu K.Q., Xiao R., Tang W.C., Wang F. (2024). The Role of p38 Mitogen-Activated Protein Kinase-Mediated F-Actin in the Acupuncture-Induced Mitigation of Inflammatory Pain in Arthritic Rats. Brain Sci..

[12] Liu F., Zhang Y.H., Zhang Y.Y., Lin J., Liu Y.J., Li Y.L., Fang Z.H., Liao H.L., Wang H., Shen J.F. (2023). Phosphorylation of the AMPARs regulated by protein kinase C (PKC) and protein interacting with C-kinase 1 (PICK1) contribute to orofacial neuropathic pain. Brain Res..

[13] Yang Y., Zhou W., Xu X., Ge X., Wang F., Zhang G.Q., Miao L., Deng X. (2021). Aprepitant Inhibits JNK and p38/MAPK to Attenuate Inflammation and Suppresses Inflammatory Pain. Front. Pharmacol..

[14] Yang B., Ma S., Zhang C., Sun J., Zhang D., Chang S., Lin Y., Zhao G. (2021). Higenamine Attenuates Neuropathic Pain by Inhibition of NOX2/ROS/TRP/P38 Mitogen-Activated Protein Kinase/NF-kB Signaling Pathway. Front. Pharmacol..

[15] Kaur A., Singh L., Singh N., Bhatti M.S., Bhatti R. (2019). Ameliorative effect of imperatorin in chemically induced fibromyalgia: Role of NMDA/NFkB mediated downstream signaling. Biochem. Pharmacol..

[16] Lin Y.W., Chou A.I.W., Su H., Su K.P. (2020). Transient receptor potential V1 (TRPV1) modulates the therapeutic effects for comorbidity of pain and depression: The common molecular implication for electroacupuncture and omega-3 polyunsaturated fatty acids. Brain Behav. Immun..

[17] Siracusa R., Paola R.D., Cuzzocrea S., Impellizzeri D. (2021). Fibromyalgia: Pathogenesis, Mechanisms, Diagnosis and Treatment Options Update. Int. J. Mol. Sci..

[18] Janssen L.P., Medeiros L.F., Souza A., Silva J.D. (2021). Fibromyalgia: A Review of Related Polymorphisms and Clinical Relevance. An. Acad. Bras. Cienc..

[19] Assavarittirong C., Samborski W., Grygiel-Gorniak B. (2022). Oxidative Stress in Fibromyalgia: From Pathology to Treatment. Oxid. Med. Cell. Longev..

[20] Farag H.M., Yunusa I., Goswami H., Sultan I., Doucette J.A., Eguale T. (2022). Comparison of Amitriptyline and US Food and Drug Administration-Approved Treatments for Fibromyalgia: A Systematic Review and Network Meta-analysis. JAMA Netw. Open.

[21] Peck M.M., Maram R., Mohamed A., Ochoa Crespo D., Kaur G., Ashraf I., Malik B.H. (2020). The Influence of Pro-inflammatory Cytokines and Genetic Variants in the Development of Fibromyalgia: A Traditional Review. Cureus.

[22] Menzies V., Lyon D.E. (2010). Integrated review of the association of cytokines with fibromyalgia and fibromyalgia core symptoms. Biol. Res. Nurs..

[23] Ghowsi M., Qalekhani F., Farzaei M.H., Mahmudii F., Yousofvand N., Joshi T. (2021). Inflammation, oxidative stress, insulin resistance, and hypertension as mediators for adverse effects of obesity on the brain: A review. Biomedicine.

[24] Liu S., Wang Z., Su Y., Qi L., Yang W., Fu M., Jing X., Wang Y., Ma Q. (2021). A neuroanatomical basis for electroacupuncture to drive the vagal–adrenal axis. Nature.

[25] Torres-Rosas R., Yehia G., Pena G., Mishra P., del Rocio Thompson-Bonilla M., Moreno-Eutimio M.A., Arriaga-Pizano L.A., Isibasi A., Ulloa L. (2014). Dopamine mediates vagal modulation of the immune system by electroacupuncture. Nat. Med..

[26] Hsiao I.H., Liao H.Y., Cheng C.M., Yen C.M., Lin Y.W. (2022). Paper-Based Detection Device for Microenvironment Examination: Measuring Neurotransmitters and Cytokines in the Mice Acupoint. Cells.

[27] Guo L., Hu H., Jiang N., Yang H., Sun X., Xia H., Ma J., Liu H. (2024). Electroacupuncture blocked motor dysfunction and gut barrier damage by modulating intestinal NLRP3 inflammasome in MPTP-induced Parkinson’s disease mice. Heliyon.

[28] Zhang C., Chen T., Fan M., Tian J., Zhang S., Zhao Z., Liu X., Ma H., Yang L., Chen Y. (2024). Electroacupuncture improves gastrointestinal motility through a central-cholinergic pathway-mediated GDNF releasing from intestinal glial cells to protect intestinal neurons in Parkinson’s disease rats. Neurotherapeutics.

[29] Lu S.F., Tang Y.X., Zhang T., Fu S.P., Hong H., Cheng Y., Xu H.X., Jing X.Y., Yu M.L., Zhu B.M. (2019). Electroacupuncture Reduces Body Weight by Regulating Fat Browning-Related Proteins of Adipose Tissue in HFD-Induced Obese Mice. Front. Psychiatry.

[30] Qin Y., He J., Xia L., Guo H., He C. (2013). Effects of electro-acupuncture on oestrogen levels, body weight, articular cartilage histology and MMP-13 expression in ovariectomised rabbits. Acupunct. Med..

[31] Yen L.T., Hsieh C.L., Hsu H.C., Lin Y.W. (2017). Targeting ASIC3 for Relieving Mice Fibromyalgia Pain: Roles of Electroacupuncture, Opioid, and Adenosine. Sci. Rep..

[32] Xie L., Liu Y., Zhang N., Li C., Sandhu A.F., Williams G., Shen Y., Li H., Wu Q., Yu S. (2021). Electroacupuncture Improves M2 Microglia Polarization and Glia Anti-inflammation of Hippocampus in Alzheimer’s Disease. Front. Neurosci..

[33] Liu B.L., Cao Q.L., Zhao X., Liu H.Z., Zhang Y.Q. (2020). Inhibition of TRPV1 by SHP-1 in nociceptive primary sensory neurons is critical in PD-L1 analgesia. JCI Insight.

[34] Kong F., Sun K., Zhu J., Li F., Lin F., Sun X., Luo X., Ren C., Lu L., Zhao S. (2021). PD-L1 Improves Motor Function and Alleviates Neuropathic Pain in Male Mice After Spinal Cord Injury by Inhibiting MAPK Pathway. Front. Immunol..

[35] Shi S., Han Y., Wang D., Guo P., Wang J., Ren T., Wang W. (2020). PD-L1 and PD-1 expressed in trigeminal ganglia may inhibit pain in an acute migraine model. Cephalalgia.

[36] Tan H., Ding Z., Zhang C., Yan J., Yang Y., Li P. (2021). The Programmed Cell Death Ligand-1/Programmed Cell Death-1 Pathway Mediates Pregnancy-Induced Analgesia via Regulating Spinal Inflammatory Cytokines. Anesth. Analg..

[37] Zhao L., Luo H., Ma Y., Zhu S., Wu Y., Lu M., Yao X., Liu X., Chen G. (2022). An analgesic peptide H-20 attenuates chronic pain via the PD-1 pathway with few adverse effects. Proc. Natl. Acad. Sci. USA.

[38] Stone J.A., Johnstone P.A. (2010). Mechanisms of action for acupuncture in the oncology setting. Curr. Treat. Options Oncol..

[39] Wang Y., Liu F., Du X., Shi J., Yu R., Li S., Na R., Zhao Y., Zhou M., Guo Y. (2024). Combination of Anti-PD-1 and Electroacupuncture Induces a Potent Antitumor Immune Response in Microsatellite-Stable Colorectal Cancer. Cancer Immunol. Res..

